# Microencapsulation technology for delivery of enzymes in ruminant feed

**DOI:** 10.3389/fvets.2024.1352375

**Published:** 2024-07-12

**Authors:** Nada Almassri, Francisco J. Trujillo, Netsanet Shiferaw Terefe

**Affiliations:** ^1^Commonwealth Scientific and Industrial Research Organisation (CSIRO), Agriculture and Food, Werribee, VIC, Australia; ^2^School of Chemical Engineering, The University of New South Wales, Sydney, NSW, Australia

**Keywords:** ruminants digestive system, undigested fibre, feed utilisation, enzyme delivery, microencapsulation

## Abstract

The ruminant digestive system is uniquely designed to make efficient use of high-fibre feed, including forages. Between 40 to 100% of the ruminant diet consists of forages which are high in fibre and up to 70% of this may remain undigested in the ruminant gut, with substantial impact on feed utilisation rate and productivity and the economic and environmental sustainability of livestock production systems. In ruminants, feed costs can make up to 70% of the overall cost of producing an animal product. Increasing feed utilisation efficiency, i.e., more production with less feed lowers feeding costs and improves livestock economic viability. Strategies for improving nutrient utilisation in animal feed has been investigated over the years. Incorporation of fibre digesting enzymes in the feed to facilitate the digestion of the residual fibre in hind gut is one of the proposed strategies. However, delivering such enzymes to the hind gut in active state is challenging due to the unfavourable biochemical environment (pH, microbial proteases) of ruminant’s gastrointestinal tract. This review discusses the potential application of microencapsulation for protected and targeted delivery of enzymes into the hind gut of ruminants.

## 1 Introduction

Ruminants include cattle, sheep, goats, deer, and other cud-chewing cloven-hoofed mammals that are poly-gastric herbivorous with a unique gastrointestinal tract that allows them to digest plant materials and makes them an important part of the food chains all over the world (1, 2). Unlike monogastric animals, the ruminant’s digestive system has one stomach with four chambers. These chambers are the rumen, the reticulum, the omasum, and the abomasum. The energy requirements of ruminant animals are met predominantly through the production of volatile fatty acids (VFAs) by the microorganisms that live in the ruminant’s stomach, mostly in the rumen, in a symbiotic relationship with the ruminant animal (3). This symbiotic relationship is beneficial to ruminants as well as the microorganisms. On the one hand, the microorganisms benefit by having a warm, wet, nutrient-rich environment to reside in. In other words, the host animal provides an anaerobic fermentation chamber for the microbes to break down the ingested feed into microbial proteins and VFAs to supply high-quality nutrients for the ruminant animal. On the other hand, a major benefit that a ruminant animal gets from the symbiotic relationship is the ability of microorganisms to digest fibre in forage-based diets (4, 5).

The enteric fermentation that takes place in the rumen is adversely correlated to environmental sustainability since it results in greenhouse gas (GHG) emissions. Several studies have been conducted to reduce ruminant methane emissions since GHG profile of the cattle dairy sector is dominated by methane emissions that is produced during the feed energy utilisation process (6). Improving ruminant productivity and energy efficiency by increasing feed digestibility, for example, is the one of the most cost-effective and promising techniques for reducing ruminant methane gas emissions (6, 7).

The digestibility of plant cell-wall material such as cellulose, hemicellulose, pectin, and lignin, as the primary source of feed for ruminants, has a significant economic implication in both developed and developing countries. However, the rumen’s ability to digest fibre is not optimum, as evidenced by the fermentability of fibre recovered from faeces (5). Ruminant animals may not digest up to 70% of their diet that contains fibrous components (8). Therefore, researchers have attempted to enhance feed utilisation rate in the rumen by manipulation of ruminal fermentation and improving diet management (7).

Ruminants lack the enzymes required to digest fibrous materials. As such, they rely on microbes within their digestive system for producing digestive enzymes (9). Therefore, most of the approaches for enhancing feed utilisation in ruminants are based on manipulation of rumen fermentation genetically or via incorporation of fibrolytic microorganisms or enzymes in ruminant feed (5). The addition of highly fibrolytic ruminal microorganisms or their enzyme extract have the potential to increase fibre digestibility and feed utilisation in ruminants (10, 11). Several *in vitro* and animal studies have been conducted on the use of exogenous enzyme formulations to improve feed utilisation and productivity in ruminants (12, 13). Nevertheless, so far, the outcomes in terms of productivity improvement are inconsistent (12, 13). This has been attributed to the use of feed enzyme formulations that are not specifically formulated for the target application, the mode of delivery of the enzymes and its effect on their stability and activity (alone or mixed with the feed, the proportion of the feed in the mix), variation in the stability of exogenous enzyme formulations in the rumen environment, and limited understanding of the rumen microbiota and their enzymes that would have enabled better design of exogenous enzyme supplements for synergistic activity with the microbial enzymes in the rumen (13).

The stability and activity of enzymes is highly dependent on environmental factors such as pH, ionic strength, temperature, and the presence of proteases and other (bio)chemicals that enhance or inhibit their activity and/or cause denaturation. Studies indicate that exogenous enzymes that bind to the feed seem to be more effective possibly due to more resistance to degradation by microbial proteases in the rumen (13, 14). The rumen gastrointestinal (GI) tract is a highly complex and harsh environment comprising of the rumen with its microbiota and their enzymes as well as the very low pH environment of the abomasum, which can negatively affect the activity of exogenous enzymes. In this respect, technologies that protect enzymes against environmental stress are of great value. Microencapsulation is one such technology that enables protection of feed enzymes against the harsh environment in the rumen and abomasum and targeted release in the GI tract for effective enhancement of feed utilisation rate.

Microencapsulation protects bioactive compounds against biotic and abiotic stresses in their environment by enclosing them in a carrier matrix (15, 16). The technology not only helps to maintain biological activity of susceptible bioactive compounds during ingestion but also enable their release at a specific location (15). Desai and Jin Park (17) described microencapsulation as a technology that was invented about 65 years ago to encapsulate liquids, gases, or solids in sealed capsules and control the release of their content under certain conditions. Several methods have been developed for producing microcapsules. Chemical methods, physicochemical methods, and physical methods are the three broad categories of microcapsule formation technologies. The method used is determined by the size of microcapsules, both the core and the wall’s chemical/physical properties, the bioactive core’s applications, economics, core sensitivity, and mechanisms of release (18, 19). Microencapsulation technology is widely used in the pharmaceutical and the food industries (15). It is also used in the ruminant feed industry to a lesser extent, to protect active compounds from rumen degradation, allowing for increased bioavailability of these compounds in the lower gastrointestinal tract of ruminants (20). A number of studies investigated microencapsulation for the delivery of actives such as essential oils (21), polyunsaturated fatty acids (22) and vitamins (23, 24) to ruminants resulting in better outcomes compared to the same amount of non-encapsulated ingredients. Microencapsulated feed supplement such as essential oils, butyrate, and bacteria are already available in the market. However, limited information is available in the public domain on microencapsulation of enzymes for post-rumen delivery in ruminants. This review briefly discusses feed utilisation efficiency in ruminants and the use of exogenous fibrolytic enzymes for improving feed utilisation and the challenges to delivery of enzymes to the ruminant GI system post rumen followed by a discussion on the potential of microencapsulation technology to overcome these challenges.

## 2 Feed utilisation in ruminants

Soluble carbohydrates as well as soluble cell wall carbohydrates such as sugars, starches, soluble fibres, pectins and β-glucans are rapidly digested in the rumen with very minimal amounts escaping to post-ruminal digestion. The majority of these soluble carbohydrates are degraded in both the small intestine and hindgut if they are not degraded in the rumen (25). However, insoluble cell wall carbohydrates, for example, neutral detergent fibre (NDF) are slowly degraded in the rumen. As a result, the retention time of the fibre in the rumen determines the extent of rumen digestion. For instance, grinding fibre into small particles may help it move through the rumen more quickly, however ruminal digestion of that fibre particle may be reduced as a result of the reduced surface area available for rumen microbes to attach and digest these fibrous materials (26). Huhtanen et al. (25) pointed out that there is no digestion of fibre in the small intestine since both soluble and insoluble fibre digestion is dependent on microbial fermentation. However, fibre that is not degraded by the rumen may be digested in the hindgut (27). However, much fewer efforts have been dedicated to understanding the digestion rate of fibre residues in the hindgut.

**Figure 1:**
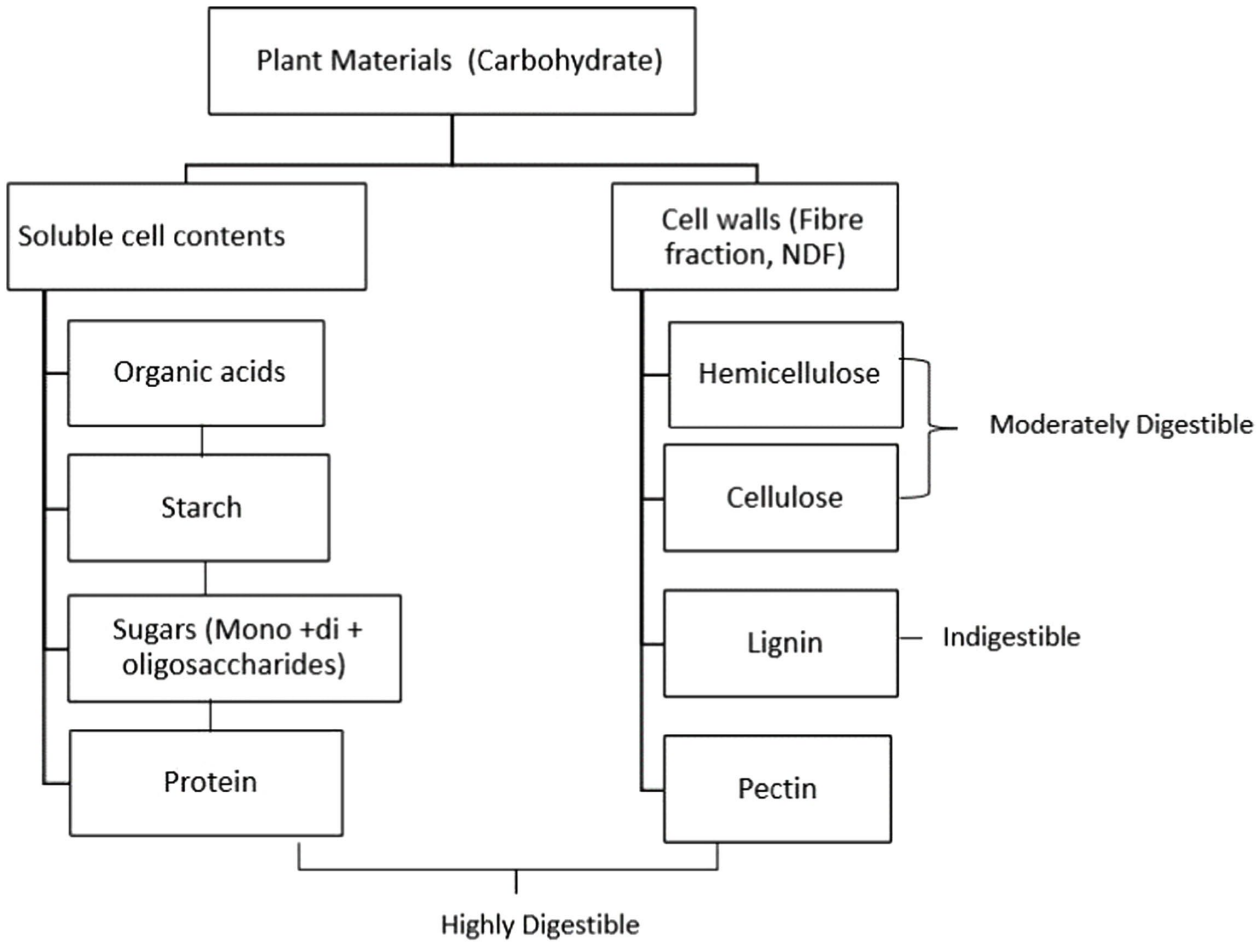
Digestibility of plant materials in the rumen.

Factors limiting fibre digestion in the rumen involve microbial enzymes such as β 1–4 cellulase enzyme complex, allowing plant cell wall polysaccharides to be hydrolysed in the rumen. However, ruminants may not digest up to 70% of their diet, particularly the fibrous components, and only 10–35 percent of energy intake is received as net energy by the ruminant animal (8). Four main factors have been identified as limiting factors for fibre digestion in the rumen: (i) animal factors that boost the nutrient’s availability through salivation, digesta kinetics, and mastication (i.e., ruminant fibre intake and mastication alter the pace of passage through the digestive tract, with higher intakes resulting in decreased total fibre digestion), (ii) microbiological factors that affect adhesion to the hydrolytic enzyme complexes of microbes in the rumen, (iii) the population of the most common fibre-digesting microbes, (iv) the biodegradability and the chemical composition of plant material’s insoluble component (28). Rumen pH appears to be a major factor in fibre digestion. A moderate drop in pH to around 6.0 causes a slight reduction in fibre digestion, but the number of fibrolytic microorganisms is unaffected. Further reduction of pH to 5.5 or 5.0 results in slowed growth rate and fewer fibrolytic bacteria, and fibre digestion may be halted. The drop in pH is caused by inadequate fibre with a high level of starch in the diet or fibre that has been chopped finely, which adversely affects chewing periods and consequently saliva production (29). Other factors such the level of lignification of the feed, and cellulolytic enzyme inhibition may affect fibre digestion (30). Kung (29) noted that lignin acts as an intercellular cement, giving plants stiffness, but reducing fermentability. According to Allen and Mertens (31), the indigestible fraction of fibre is the most significant independent constraint on fibre digestion. This fraction makes up 1/3 to 1/2 of the total fibre fraction. The digestibility of various plant components in the rumen is illustrated in Figure 1.

## 3 Approaches for improving feed utilisation in ruminants

Improving fibre digestion in ruminants has been a focus of research in rumen microbiology for several decades. Ruminants rely on the microbial population within their rumen to produce the necessary enzymes for fibre digestion, as they lack the required endogenous enzymes themselves (9). The supplementation of highly fibrolytic ruminal microorganisms or their enzyme extracts has been found to increase fibre degradability in ruminants (10). However, the effects on growth traits and feed efficiency ratio were not constant, and the impact may vary depending on breeds and dosages used. Further research may be required to assess the optimal dosage and specific benefits of exogenous fibrolytic enzymes supplementation in ruminants’ nutrition.

Other factors such as diet composition, feed processing methods, environmental stress and rumen microbial populations can also influence fibre digestion and feed utilisation in ruminants. For instance, diets containing high levels of readily fermentable carbohydrates may impede the efficacy of enzymes in fibre digestion (32). Therefore, a comprehensive understanding of the interactions between enzymes, microorganisms, and the rumen environment is necessary to optimise the use of enzymes for enhancing feed utilisation.

### 3.1 Enzyme supplementation for enhancing feed utilisation in livestock

The digestive efficiency of ruminants is not optimal, and a significant portion of feed remains undigested due to indigestible anti-nutritional factors or the absence of specific enzymes (33). Feed enzyme additives primarily target the fibrous fraction of forages, with limited exploration of amylases for starch utilisation (33–35). Forages typically contain 30–70% neutral detergent fibre (NDF) on a dry matter (DM) basis. Despite optimal feeding conditions, the digestibility of NDF in the digestive tract of ruminants tends to be below 65%, and ruminal NDF digestibility is often less than 50%. As feed constitutes a substantial cost in animal production, supplementing it with specific enzymes enhances the nutritional value of ingredients, improving digestion efficiency and overall animal performance (33). The supplementation of animal feeds with exogenous enzymes has been successfully practiced since the late 1980s (36). Today, the overall value of the feed enzyme market has expanded approximately fourfold compared to its early 2000s valuation. Table 1 provides a comprehensive review on enzyme additives for ruminants endeavouring to articulate a well-founded rationale for their efficacious integration into both beef and dairy diets.

**Table 1:** Summary of relevant studies on the use of exogenous enzymes for enhancing feed utilisation in ruminants.

Study	Enzyme used	Supplemented diet	Type of study	Exogenous enzymes application	Outcome	References
**1**	Commercial source of cellulase enzyme (CENZ) Cellulase enzyme cocktail produced in-farm (FENZ) using *Penicillium Chrysogenum.*	Total mixed ration (TMR)	*In vivo*	Lactating buffaloes	**Nutrient Digestibility:** FENZ demonstrated significantly higher dry matter digestibility compared to CENZ. FENZ supplementation had a notably higher impact on crude protein (CP), neutral detergent fibre (NDF), acid detergent fibre (ADF) and ether extract (EE) digestibility compared to CENZ. **Daily Milk Yield and Fat-Corrected Milk Yield (FCM):** Supplementing the diet with any cellulase enzyme source resulted in a noteworthy increase in both daily milk yield and fat-corrected milk yield (FCM). The FENZ group exhibited significantly higher daily milk yield and FCM compared to CENZ.	(37)
**2**	Xylanase 1 (XY1), 3 (XY3) or 6 (XY6) μl/g	Basal diet	*In vivo*	Rambouillet sheep	**Feed Intake:** Sheep fed XY1 and XY3 demonstrated significantly higher feed dry matter (DM) intake compared to control sheep, with increases of 6 and 3% for XY1 and XY3, respectively. However, at the highest application rate (XY6), feed intake slightly decreased when compared with the control group. **Digestibility:** Sheep fed XY1 and XY6 exhibited significantly greater total tract digestibility of DM, organic matter (OM), and crude protein (CP) compared to the control group. Dry matter digestibility notably increased by 30% with XY6 in comparison to the control diet. **Ruminal ADF & NDF Digestibility:** Sheep fed the enzyme exhibited significantly higher ruminal neutral detergent fibre (NDF) digestibility compared to the control group. However, no significant difference was observed in ruminal acid detergent fibre (ADF) digestibility between sheep fed the enzyme and those on the control diet.	(38)
**3**	Cellulase Xylanase A combination of both	Forage-to-concentrate ratio (F:C) ratio.	*In situ, in vivo or in vitro*	Sheep, lactating dairy cows and beef cattle	**Dairy Cows:** In low-forage diets (F:C < 50%), supplementation with exogenous fibrolytic enzymes (EFE) did not yield positive effects on milk production and milk solid contents. EFE application to high-forage diets (F:C ratio ≥ 50%) positively impacted milk production, with significant increases in milk protein (1.96 kg/d and 99.44 g/d) and milk fat (83 g/d). EFE supplementation negatively affected dry matter intake (DMI) across all diets. **Beef Cattle:** In diets with low-forage concentration (<50% grasses), EFE supplementation led to an increase in average daily gain (ADG) by 0.30 kg/d. Positive effects on feed conversion (FC) were observed in low-forage diets (F:C < 50%) with EFE treatment. Although improvements in DMI were seen in diets with ≥50% grasses there were no positive effects on ADG and FC when EFE was used as a supplement in high-forage diets (F:C ≥ 50%).	(39)
**4**	Exogenous fibrolytic enzymes: Endo 1,4- β glucanase 1 (3)-4-β glucanase Endo 1,4-β xylanase	Total mixed ration (TMR)	*In vivo*	Holstein Friesian [HF] crossbred cows.	**Body Weight and Nutrient Intake:** Non-significant differences observed in average body weight changes and nutrient intake between control TMR (T1) and EFE-supplemented TMR (T2). EFE supplementation at 240 mg/kg TMR improved daily intake of dry matter (DM), crude protein (CP), digestible crude protein (DCP), and total digestible nutrients (TDN). **Milk and Components Yield:** Cows fed EFE-supplemented TMR produced significantly more milk (12.34%) and 4% fat-corrected milk (FCM) yield (15.70%) compared to those on control TMR. Improved milk fat, solid-not-fat (SNF), and total solids (TS) contents were observed in EFE group. **Feed Conversion Efficiency and Cost of Feeding:** Numerical improvements in DM, CP, DCP, and TDN intake to produce each kg of whole milk in EFE-supplemented TMR. Gross protein and energetic efficiency improved significantly with EFE supplementation, leading to higher returns over feed cost. **Economic Returns:** The improved ratio of income over feed cost was attributed to enhanced energy availability and efficient nutrient utilisation.	(40)
**5**	Fibrolytic enzyme (FIB), Fibrozyme Amylolytic enzyme (AMY), Amaize A combination of both FIB+AMY	Basal Diet	*In vivo*	Dairy cows in mid-lactation.	**Dry Matter (DM) and Nutrient Intake:** Treatments had no significant effect on DM and nutrient intake **Milk Yield and Composition:** No effect on yields of milk, fat-corrected milk (FCM), and milk fat. Fibrolytic enzyme tended to decrease milk protein concentration, especially in the absence of AMY. Interaction effect (*p* ≤ 0.053) between FIB and AMY observed for lactose and protein production, and feed efficiency. **Ruminal Parameters:** No significant effect of enzymes on ruminal pH, NH3-N concentration, acetate, propionate, branched-chain fatty acids, and volatile fatty acids (VFA). **Nitrogen Utilisation:** No significant effects on N intake, faecal excretion, microbial protein synthesis, serum glucose, urea concentration, and milk N secretion. Amylolytic enzyme decreased urinary N excretion, without affecting N balance.	(41)
**6**	Accellerase®XC	Two proportions of oat silage to concentrate ratios: (AS-60) (AS-70)	*In vivo*	Early lactating buffaloes	**Dry Matter (DM) Intake:** No significant difference in DM intake between groups with or without EFE supplementation. However, EFE-supplemented groups showed a higher intake of DM, crude protein (CP), neutral detergent fibre (NDF), and acid detergent fibre (ADF) as a percentage of body weight and on a metabolic body weight basis. The AS-60 group had the highest digestible nutrient intake, followed by AS-70, compared to groups without EFE. **Nutrient Digestibility:** Buffaloes in the EFE-supplemented groups (AS-60 and AS-70) demonstrated improved digestibility of DM, CP, NDF, and ADF compared to those without EFE. **Blood Urea Nitrogen and Glucose:** Blood urea nitrogen and glucose levels remained within the normal range, showing no significant differences among dietary treatment groups. **Milk Production and Composition:** Significantly better milk production in animals fed enzyme-treated diets compared to those without enzyme supplementation. Milk fat, total solids, and milk energy content increased in animals fed AS-60 and AS-70 diets. No significant differences in milk protein, lactose, and solid-not-fat content. AS-60 and AS-70 diets exhibited better feed conversion (DM or CP intake to produce one kg of 4% FCM) compared to other diets.	(42)
**7**	Exogenous enzymes (EZ) *Saccharomyces cerevisiae* (SC) A combination of both EZSC	Basal diet	*In vivo*	Goats	**Feed Intake:** Significantly higher feed intake observed in groups receiving EZ, SC, and EZSC treatments compared to the control group. No significant differences observed among the EZ, SC, and EZSC treatments. **Milk Yield and Efficiency:** EZ, SC, and EZSC treatments resulted in significantly milk yield, energy-corrected milk (ECM) yield, milk component yields, and milk energy output compared to the control group. **Milk Composition:** Higher concentrations of milk total solids, solids-not-fat, lactose, and milk energy observed in groups receiving EZ, SC, and EZSC treatments. **Feed Efficiency:** The treatments involving EZ, SC, and EZSC showed enhanced feed efficiency, as indicated by greater milk yield and energy output per unit of feed consumed.	(43)
**8**	Cellulase Xylanase	(55% rice straw, 45% concentrate)	*In vivo*	Goats	**Intakes and Digestibility:** No significant differences observed in the intakes and apparent digestibility of organic matter (OM), neutral detergent fibre (NDF), and acid detergent fibre (ADF) among treatments. **Faecal Outputs and Total Tract Digestibility**: Addition of exogenous cellulase and xylanase did not influence faecal outputs and apparent total tract digestibility of OM, NDF, and ADF **Gross Energy, Faecal Energy, and Urinary Energy:** No differences in daily ingested gross energy (GE), faecal energy (FE), and urinary energy (UE) among all treatments. **Ruminal Fermentation:** No significant effects on concentrations of NH3-N, total volatile fatty acids (VFA), molar proportions of acetate and propionate, and the acetate to propionate ratio in the rumen of goats.	(44)
**9**	Promote®	35% Tifton 85 bermudagrass silage 10% Corn silage 55% Concentrate Total mixed ration (TMR)	*In vivo*	Lactating dairy cows	**Voluntary Intake and Apparent Digestibility:** Enzyme addition did not affect dry matter (DM), neutral detergent fibre (NDF), or crude protein (CP) intake. Lack of intake response attributed to unaffected digestibility. **Milk Production and Composition:** Cows fed the control diet tended to produce more milk than those fed enzyme-treated concentrate (EC). EC resulted in lower ruminal pH, potentially contributing to reduced milk production. Inconsistency in milk production responses compared to other studies. **Ruminal pH and Concentrations of VFA and NH3-N:** Lower NH3-N concentration in cows fed enzyme-treated total mixed ration (ETMR), suggesting enhanced NH3-N uptake by ruminal microbes. Lower total volatile fatty acid (VFA) concentration in cows fed ETMR, enzyme-treated forage (EF), and enzyme-treated silage (ES) compared to the control diet. *In Situ***DM Disappearance:** Results for ETMR, EF, and EC diets agreed with *in vivo* digestibility results, showing no effects on forage degradability.	(45)
**10**	A combination of Xylanase and endoglucanase exogenous enzyme product (Roxazyme®)	Sorghum or barley grain-based diets	*In vivo*	Beef steers	**Voluntary Dry Matter (DM) Intake:** Increased daily voluntary DM intakes observed for steers fed the sorghum diet with EE treatment. No significant impact on DM intake for steers fed the barley diet. **Live Weight (LW) Gain:** Numerical increase in daily LW gain observed on both diets with no significant changes in feed efficiency.	(46)
					**Digestibility:** No effect of EE treatment on total tract organic matter (OM) or fibre digestibility. Interaction with diet observed, where sorghum starch digestibility was reduced by EE treatment, while barley starch digestion remained unchanged. **Urinary Nitrogen (N) Excretion:** EE supplements increased urinary N excretion.	
**11**	β-mannanase	40% of tall fescue hay +60% of concentrate mix	*In vivo*	Goats	**Dry Matter (DM) Intake:** No significant differences observed among treatments. **Average Daily Gain (ADG) and Feed Conversion Ratio (FCR):** Goats fed diets with β-mannase showed significantly higher ADG and lower FCR compared to the control group. ADG tended to increase linearly with the dosage of β-mannase supplementation. **Digestibility:** DM and Organic Matter (OM) digestibility were significantly greater for goats fed the basal diet without enzyme supplementation compared to those with β-mannase supplementation. **Protein and Fibre Digestibility:** No significant effects of enzyme addition on Crude Protein (CP) and Neutral Detergent Fibre (NDF) digestibility among treatments. **Nitrogen (N) Intake and Retention:** N intake and retention increased significantly with increasing dosage of β-mannase supplementation.	(47)

The reported outcomes from various studies demonstrate the potential of enzyme supplementation on rumen function, nutrient digestibility, milk production, and growth performance. However, it should be noted that the outcomes of using exogenous fibrolytic enzymes (EFE) is so far inconsistent, and their activity and efficiency varies depending on the type and quality of the diet and animal species. For example, the studies by Lee et al. (47), Zilio et al. (41) and Miller et al. (46) collectively contribute diverse insights into the complex effects of exogenous enzyme supplementation in ruminants. Lee et al. (47) and Zilio et al. (41) share a common observation, revealing no significant impact on dry matter (DM) intake with enzyme supplementation. However, Miller et al. (46) diverges by noting an increased intake, specifically in the sorghum-fed group, underlining the influence of both enzyme type and dietary composition. Furthermore, while Zilio et al. (41) and Miller et al. (46) converge in reporting no significant impact on overall digestibility with enzyme treatment, Lee et al. (47) presents a unique finding of reduced digestibility with β-mannase supplementation, emphasising the need to consider specific enzymes and substrates in understanding the broader implications. Lastly, while Zilio et al. (41) and Miller et al. (46) do not find significant effects on milk yield and composition, Lee et al. (47) leaves this aspect unexplored, highlighting the potential for a more comprehensive understanding by integrating studies that consider both animal performance and milk production outcomes. One of the reasons for the inconsistent results is the susceptibility of enzymes to proteolytic degradation by rumen microorganisms (39, 48). Enzymes can be degraded by proteases present in the rumen, limiting their effectiveness for enhancing fibre digestion. The divergence in outcomes has been ascribed to various factors, including the enzyme formulation, enzymatic activity, dosage level, mechanism of action, supply method, stability of the enzymes within the rumen, the overall composition of the diet and experimental conditions (41). This variability in enzyme activity and degradation highlights the complexity of the rumen ecosystem and the challenges associated with utilising exogenous enzymes as a reliable approach for improving fibre digestion.

Further research is needed to explore strategies that can enhance the stability and effectiveness of exogenous enzymes in the rumen. This may involve the development of enzyme formulations that are more resistant to proteolytic degradation or utilising enzyme delivery systems that can protect enzymes until they reach the site of action in the gastrointestinal tract of ruminants. Nonetheless, the use of enzymes represents a promising approach to maximise feed utilisation and improve the efficiency of ruminant production systems. These advancements will contribute to the development of more effective and reliable approaches for enhancing fibre digestion and optimising feed utilisation in ruminant production systems.

### 3.2 Challenges for delivery of enzymes to ruminant gut

**Figure 2:**
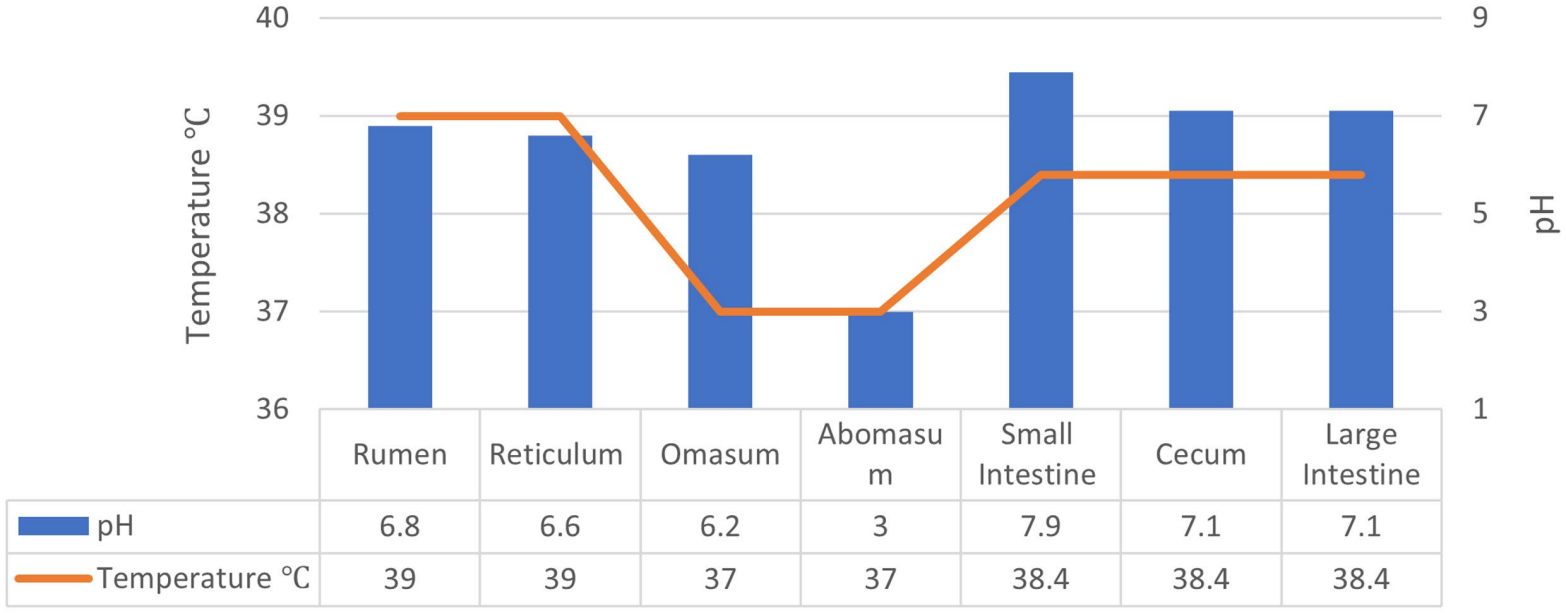
The environmental conditions across the GIT of ruminants.

As discussed earlier, the application of exogenous fibrolytic enzymes has a significant potential for improving feed utilisation efficiency in ruminants. However, delivering enzymes in active form to the rumen and beyond in the GI tract of ruminants is quite challenging especially due to the susceptibility of enzymes to environmental factors such as pH as well as proteolytic degradation. The temperature and pH conditions in the GI tract of ruminants is summarised in Figure 2. The rumen houses a highly complex and diverse microbiota and is considered as a “fermentation vat” since it contains microbial communities that feed on forages and produce the enzymes required to degrade the components of plant cell walls such as hemicellulose, cellulose, pectin, and lignin as well as proteases (3, 26). Thus, exogenous enzymes may not maintain their activity and contribute to fibre digestion in the rumen as they may be degraded by proteases. The abomasum with its very low pH and pepsin presents another challenging environment to exogenous enzymes so do trypsin in the small intestine and the microbiota and their enzymes in the hind gut.

One way of overcoming this challenge is the use of microencapsulation, i.e., enclosing the enzyme in a carrier matrix that protect it against environmental stress and enable its targeted delivery at a specific location within the GI tract. Microencapsulation helps to protect the encapsulated core material (enzyme or other active ingredients) against ruminal degradation by lowering its reactivity or transference to the outside environment, and/or from the unfavourable environmental conditions such as pH, and microbial communities, resulting in increased bioavailability of the active ingredient in the lower GI tract of ruminants (20, 49). Microencapsulation is effective in ensuring rumen by-pass of active ingredients and their bioavailability (50). For instance, a study by Yoshimaru et al. (51) showed that spray dried microcapsules of proteases have successfully passed through rumen with little degradation and their content was released in the abomasum to increase protein absorption in the intestines. The success of the microencapsulation process depends on the appropriate selection of the wall materials and encapsulation method, that protect the active core and allow it to be released in active form at the desired location (52–54). This innovative approach not only protects the encapsulated core material, such as enzymes, from environmental stress but also ensures their targeted delivery, ultimately leading to increased bioavailability in the lower gastrointestinal tract of ruminants.

## 4 Microencapsulation techniques for enhancing stability and controlled release of enzymes

In various industrial sectors where enzymes play a pivotal role, the issue of enzyme stability emerges as a crucial obstacle in optimising their efficacy. Despite the potential benefits of exogenous enzyme supplementation in improving fibre digestibility and nutrient absorption, the practical application of these enzymes is frequently impeded by challenges pertaining to their stability (39, 41, 48). Roy Choudhury (55) stated that enzymes are most effective when the pH and temperature are maintained within a limited range. Based on that, the shape of the active site of an enzyme can be altered by heating or changing the pH of the enzyme’s surroundings, rendering it inactive. Factors such as extreme pH and temperature variations, exposure to proteolytic enzymes, and interactions with dietary components can lead to enzyme denaturation, degradation, or loss of activity before reaching their intended targets (56–59). Thus, there is a critical need for research and development efforts aimed at enhancing enzyme stability across various environments. Addressing these stability issues is paramount to unlocking the full potential of enzyme supplementation. Thus, microencapsulating these enzymes is a viable option for improving their application in commercial products and industrial processes (60).

**Figure 3:**
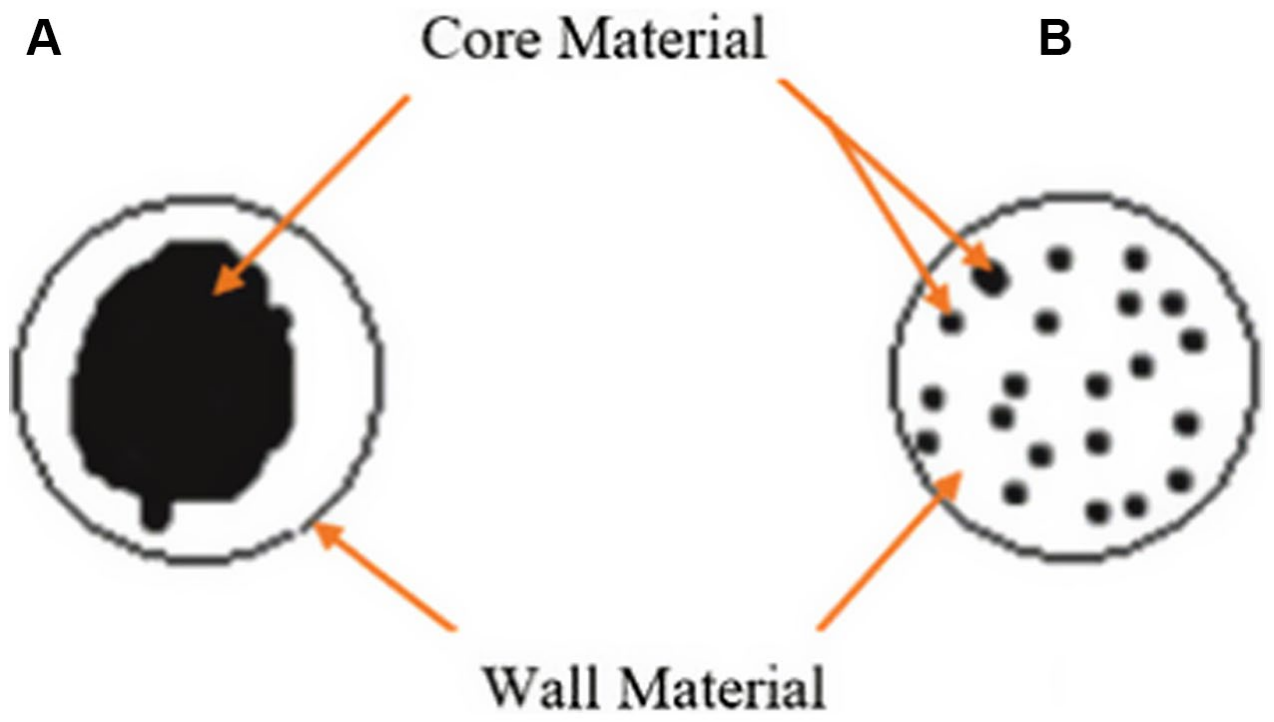
Schematic diagrams of two representative types of microencapsulation preparations, **(A)** Microcapsule, **(B)** Microsphere (63).

Microencapsulation is a versatile technique that has been widely utilised for the encapsulation of various compounds, including water-soluble substances like enzymes. This process involves the coating or surrounding of individual particles, referred to as core or active materials, with a protective wall material, resulting in the formation of microcapsules or microspheres (61, 62). Microcapsules consist of an inner core containing the enzyme and a polymer membrane that encapsulates it, while microspheres are composed of a polymer matrix with the enzyme uniformly dispersed or dissolved within it as shown in Figure 3 (64, 65). Microencapsulation has emerged as a promising technique for the protected delivery of enzymes to the gastrointestinal (GI) tracts of both humans and animals, including poultry (62, 66). This innovative approach involves encapsulating enzymes within protective coatings, allowing for controlled release and targeted delivery within the GI environment (67).

The primary objective of microencapsulation is to protect the encapsulated enzyme from harsh environmental conditions, such as pH, temperature, and oxidative stress, thereby enhancing its stability and prolonging its shelf life (54). The encapsulation process creates a barrier between the enzyme and its surroundings, preventing degradation and maintaining its activity during storage or when exposed to unfavourable conditions. Table 2 shows a summary of studies on microencapsulation techniques for enzyme protection against environmental stress.

**Table 2:** Summary of relevant studies on microencapsulation techniques for enzyme protection against environmental stress.

Enzyme	Microencapsulation material	Protection mechanism	Application	Outcome	Reference
α-amylase	Beeswax	Acts as a physical barrier and provides stability against pH and temperature	Used in gluten-free bread production in the food industry	Encapsulated α-amylase enzyme exhibited improved sensorial quality and stability during a storage period of 5 days	(68)
Lactase	PLGA-PEG	Nanoparticles against vascular oxidative stress	Antioxidant enzyme carrier systems	The nanoparticles exhibited considerable enzyme activity retention of approximately 40% after a 5-h exposure to proteolytic conditions without causing oxidative stress or cytotoxic effects.	(69)
Lipase	γ-(methacryloxypropyl)-trimethoxy silane (MAPTMS)	Provides thermal stability to the encapsulated lipase	Widespread applications	The lipase encapsulated within the matrix demonstrated superior thermal stability, particularly at elevated temperatures of 50°C and 60°C, when compared to the free form. It retained 54% of its initial activity even after 60 days of use, while the free enzyme exhibited a decrease to 52% activity after only 30 days.	(70)
Lactase	Double-capsule delivery system composed of enteric-coated capsule and PLA nanocapsules	Protection against enzymatic degradation	Lactose hydrolysis within the gastrointestinal tract	Lactase-loaded PLA nanocapsules achieved almost complete hydrolysis of lactose in milk, while free lactase only achieved approximately 61.7% hydrolysis after 180 min of incubation. This demonstrates the efficient hydrolysis of lactose in the gastrointestinal tract using lactase-loaded PLA nanocapsules, highlighting their potential for effective oral delivery of other proteins with high bioavailability	(57)
Lactase	Carrageenan hydrogel beads	Enhanced enzyme activity across a wide range of pH and thermal conditions.	Food and pharmaceutical industries.	Encapsulated β-galactosidase (lactase) showed higher activity than the free enzyme, attributed to the stabilisation of the enzyme structure by K+ ions within the carrageenan beads	(71)
Catalase	Polyelectrolyte multilayer capsules on biocrystal templates	Protects against protease degradation	Drug delivery applications.	Polymer-coated catalase retained 100% of its activity after incubation with protease, while uncoated catalase lost more than 90% of its initial activity within the same period	(56)
Catalase	Filamentous polymer nanocarriers (*f*-*PNC*)	Provides resistance to protease degradation	Therapeutic applications.	The percent protection of the protein against protease degradation, measured as the amount of protein resistant to degradation relative to the total loaded protein in filamentous polymer nanocarriers (*f-PNC*), varied from 15 to 29%, which was significantly higher compared to non-PNC encapsulated catalase preparations with protection levels less than 5%.	(58)
Protease	Silica nanocapsules using an amphiphilic precursor polymer (PEG-PEOS)	Protects against changes in environmental conditions	Development of biosensors, drugs, and enzyme reactors,	Compared to the free enzyme, the encapsulated protease demonstrates a preservation of approximately 40% of its activity, along with significantly improved stability against variations in temperature, pH, and the concentration of chaotropic surfactants. Furthermore, the encapsulated protease exhibits the ability to be regenerated multiple times without a notable loss of activity.	(59)
Superoxide dismutase (SOD)	Combination of Liposomes and Iontophoresis	Provides protection against UV radiation	Non-invasive transfollicular delivery system for macromolecules	Iontophoretic delivery of liposomes containing SOD reduced the production of oxidative products in UV-irradiated skin, demonstrating the potential of the delivery system for other macromolecules	(72)

One of the significant advantages of microencapsulation is its ability to improve the stability of enzymes. Studies have shown a significant increase in the thermal stability of encapsulated enzymes compared to their free counterparts. For example, microencapsulation of phytase by alginate beads was reported to significant increase the stability of the enzyme from 20 to 79% (73). In Another study conducted by Zhao et al. (74), lipase encapsulation resulted in the preservation of 87.5% of its activity following exposure to a thermal treatment at 70°C for 2 h, whereas the unencapsulated enzyme experienced a substantial loss of 99.5% activity under identical conditions. Similarly, Yang et al. (70) noted that lipase encapsulated within a matrix retained higher activity at elevated temperatures (50°C and 60°C) for an extended period compared to the free form. This enhanced thermal stability is attributed to the protective effect of the encapsulating material, which acts as a shield against heat-induced denaturation and degradation. Furthermore, microencapsulation provides a buffer against pH variations, maintaining enzyme activity over a broader pH range. Zhang et al. (59) found that encapsulated protease retained approximately 40% of its activity under varying pH conditions, indicating improved stability compared to the free enzyme. This stability is crucial for applications in diverse pH environments, such as industrial processes or gastrointestinal delivery. Additionally, Free enzymes are susceptible to proteolytic degradation, limiting their functionality in biological systems. However, encapsulation offers protection against proteases, as observed by Sari et al. (69) and Simone et al. (58). These studies reported significant enzyme activity retention even after exposure to proteolytic conditions, highlighting the potential of encapsulation in enhancing enzyme stability in biological fluids or environments rich in proteases.

In addition to improving stability, microencapsulation offers several other benefits for enzymes. It allows controlled release of the encapsulated enzyme, enabling targeted delivery to specific sites or controlled release over a prolonged period (75, 76). This controlled release is particularly advantageous in applications where a sustained enzyme activity is required, such as in drug delivery systems or in biotechnology industries. The key parameters influencing release rates are interactions between the wall material and the core, core’s volatility, the ratio of the core to the wall material, and the wall material’s viscosity grade (77). The primary mechanisms involved in core release are shown in Table 3. More than one mechanism is used in practice (17).

**Table 3:** The primary mechanisms involved in core release.

pH	Variations in pH can affect the solubility of the wall material, allowing the core to be released. For instance, enzymes can be microencapsulated to withstand the acidity of the stomach and released only when the intestine is alkaline.
Temperature	Temperature changes can induce core release by two different mechanisms: Temperature-sensitive release, which occurs when a critical temperature is reached, is allocated for expanding or collapsing materials, whereas fusion-activated release entails melting of the wall material owing to temperature increase.
Solvent use	When the wall material comes into touch with a solvent, it can dissolve completely, releasing the core rapidly, or it can expand.
Diffusion	Diffusion happens most often when the microencapsulate’s wall is intact, the chemical properties of the core and wall material, as well as several physical parameters of the wall, as well as environmental factors determine the release rate.
Pressure	When a force is applied to the microencapsulate wall, it causes the core to be released such as when chewing gum is masticated, some flavours are released.
Degradation	Degradation occurs when enzymes such as lipase and protease break down lipids or proteins.

Furthermore, microencapsulation provides a means to enhance the loading capacity of enzymes. The encapsulating material can accommodate a high concentration of enzymes, maximising their content within the microcapsules or microspheres. This high enzyme loading capacity ensures that a significant amount of active enzyme is available for the desired application. For example, the successful encapsulation of Flavourzyme^®^ into a cross-linked chitosan matrix enabled improved loading capacity of Flavourzyme^®^ in chitosan-TPP microparticles and could be a promising option for utilising commercial proteases in food processing (78). Thomas et al. (79) and Anjani (80) highlighted the efficacy of a microcapsule system in facilitating the efficient loading of enzymes while ensuring extended shelf life and sustained activity. Moreover, Weng et al. (73) successfully optimised the overall efficiency of phytase encapsulation by employing a three-fluid nozzle spray drying technique, resulting in a substantial enzyme loading capacity of 48 wt%. These findings collectively highlight the potential of different encapsulation strategies in enhancing enzyme loading and preserving their activity for diverse applications in various fields.

Overall, microencapsulation has emerged as a promising approach for the effective delivery and protection of water-soluble compounds, including enzymes. The technique offers improved stability, controlled release, and enhanced loading capacity, making it a valuable tool in various fields such as pharmaceuticals, biotechnology, food, and cosmetics. Further research and development in microencapsulation methods and materials hold great potential for advancing the application of enzymes in diverse industries.

### 4.1 Selection of encapsulating methods and wall materials

Microencapsulation techniques are classified into three groups: (i) physicochemical methods such as thermal gelation, coacervation (phase separation), and emulsification, (ii) chemical methods including polymerisation and interfacial polycondensation, (iii) physical (mechanical) methods like solvent evaporation, spray drying, fluidized bed coating, centrifugation, and extrusion (81, 82). Blaine (52) listed five techniques that are often employed in the production of micro and nanoparticles, including fluidized bed coating, spray freezing, coacervation, extrusion, and spray drying. The most appropriate encapsulation method for a given application is determined by the core type, the microcapsule’s application, the particle size required, chemical and physical features of the wall and core materials, the required release mechanism, cost, and production scale (52, 54). There are several studies in the literature on the microencapsulation of various active compounds for food, feed, cosmetic and pharmaceutical applications. This literature review is focused on microencapsulation techniques that are relevant to water soluble actives such as enzymes.

The Encapsulating materials should be chosen carefully due to their impact on the capsule’s stability and efficiency. The choice of the encapsulating materials is considered as one of the most important and first steps in the development of encapsulation techniques. The materials are selected based on the active component, the core material, and the desired characteristics of the final product (53). The wall material should have the following characteristics to be successfully delivered to the desired location: (i) not reacting with the core, (ii) ability to keep the core sealed and stable inside the capsule, and (iii) ability to give optimum protection to the core against unfavourable circumstances (18, 19, 76, 83). To protect the core materials from ruminal degradation, wall materials should have the following specific properties: (i) be insoluble in an animal’s rumen and abomasum with a pH greater than 6.0 and less than 2.0 respectively, (ii) be able to withstand microbial attack, (iii) have mechanical qualities that allow them to endure breakage (20). However, as reported by Desai and Jin Park (17) most wall materials lack all of the desired qualities. Thus, a frequent approach involves combining two or more wall materials. Other essential properties to consider are cost-effectiveness and suitability and safety for feed application.

## 5 Microencapsulation application in the ruminant feed industry

In animal nutrition, microencapsulation can be utilised for a variety of applications. It can be used to deliver substances to a specific location of the gastrointestinal (GI) tract (i.e., targeting a particular site of the intestinal tract as a dietary component of the feed), to mask the flavour of products with undesirable flavour, to ensure product stability during long periods of storage, or to increase the bioavailability of a product to improve animal performance (84). Several studies have contributed to refining methods for preserving nutrient integrity during ruminal passage. Yoshimaru et al. (51) demonstrated the successful transit of spray-dried microcapsules of protease through the rumen with minimal degradation, releasing their content in the abomasum. Similarly, studies by Yoshimaru et al. (85) highlighted the potential of spray-dried L-lysine with zein to resist ruminal damage and pass through the rumen with minimal degradation. Recent investigations, such as (86), have explored microencapsulation with ethyl cellulose and carbomer to protect L-carnitine in the rumen, showcasing improved antioxidant capacity compared to unprotected supplementation. Likewise, encapsulation of soybean meal with zein or wheat gluten demonstrated enhanced ruminal protection compared to unprotected soybean meal (52).

The field has seen diverse approaches, including studies comparing microencapsulation methods and wall materials for nutrient protection. Chauhan and Gautam (87) assessed pan coating and fluidized bed coating methods, determining that choline chloride granules coated with cellulose-based polymer and hydrogenated vegetable oils using fluidized bed coating exhibited high rumen bypass potency. Conversely, Pretorius (88) found that evaluated wall materials like zein and kafirin displayed consistent release curves during ruminal transit, with protein coatings failing to provide complete protection. Chiang et al. (89) and Cao et al. (90) delved into the stability of protected amino acids and the controlled release of L-carnitine, emphasising the importance of coating composition and levels. Other authors have also highlighted the coating levels influenced the efficacy of active ingredients release at the desired location (86, 91–93). Notably, Ibrahim and Hassen (67) employed the solid-in-oil-in-water (S/O/W) technique to encapsulate Mimosa tannin, achieving smaller diameters, lower density, and high encapsulation efficiency. The study demonstrated controlled release dynamics, with a fraction released in the stimulated rumen, and specific percentages in the abomasum and small intestine.

Furthermore, Carrillo-Díaz et al. (94) stated that understanding the efficacy of encapsulated materials necessitates a consideration of various factors, including enzyme type and dose, forage composition, and the forage-to-concentrate ratio. *In vitro* results may not perfectly align with *in vivo* effects, highlighting the intricate interplay of exogenous fibrolytic enzymes (EFEs), ruminal fluid, and animal-specific conditions. In conclusion, numerous studies underscore the potential of microencapsulation in delivering various compounds to distinct regions of the ruminant GI tract. Despite the complexities involved, these endeavours collectively contribute to advancing our understanding of microencapsulation’s feasibility and efficacy in optimising nutrient delivery for improved animal nutrition and well-being.

## 6 Conclusion and future perspectives

Low feed utilisation efficiency is still a major problem in livestock production with forage as the main feedstock. Various approaches such as breeding and genetic approaches for breeds with higher feed utilisation efficiency and supplementing the feed with exogenous microorganisms and fibre-digesting enzymes have been evaluated for improving feed utilisation efficiency with different degree of success. The use of exogenous enzymes in particular has significant potential for enhancing fibre digestibility in rumen and post-rumen. However, better enzyme formulations to synergistically work with rumen microbial enzymes and delivery systems that protect exogenous enzymes during delivery to the rumen and beyond in the GI tract of ruminants are required in order to fully benefit from enzyme supplementation. In this regard, the use of microencapsulation for protected and targeted delivery of enzymes has a huge potential. Improving feed utilisation rate in ruminants, will result in increasing production of milk and meat for human consumption and improving the efficiency of livestock production in a more cost-effective manner as well as reducing environmental footprint. Although many studies and reviews on the delivery of encapsulated active compounds in ruminants have been undertaken, it is still unknown if the delivery of encapsulated enzymes in active form to the rumen and the hindgut of ruminants would be successful. Therefore, further studies are needed to develop microencapsulation approaches that enable the delivery of enzymes at specific targets in the GI tract of ruminants. Additionally, studies evaluating the stability of encapsulated enzymes under simulated gastrointestinal conditions, such as variations in pH, temperature, and enzymatic activity are needed. Assessing the bioavailability and efficacy of encapsulated enzymes *in vivo* is essential to determine their impact on fibre digestion and feed utilisation efficiency in ruminants. Investigating the long-term effects of microencapsulated enzyme supplementation on animal health, productivity, and performance is also critical for ensuring safety and sustainability. These studies will help refine microencapsulation approaches for enzyme delivery in ruminants, ultimately enhancing fibre digestion, feed utilisation efficiency, and livestock productivity in a sustainable manner.

## Author contributions

NA: Visualization, Conceptualization, Investigation, Writing – original draft, Writing – review & editing. FT: Funding acquisition, Resources, Supervision, Writing – review & editing. NT: Conceptualization, Funding acquisition, Resources, Supervision, Writing – review & editing.
